# Synthetic Spike-in Standards Improve Run-Specific Systematic Error Analysis for DNA and RNA Sequencing

**DOI:** 10.1371/journal.pone.0041356

**Published:** 2012-07-31

**Authors:** Justin M. Zook, Daniel Samarov, Jennifer McDaniel, Shurjo K. Sen, Marc Salit

**Affiliations:** 1 Biochemical Science Division, National Institute of Standards and Technology, Gaithersburg, Maryland, United States of America; 2 Statistical Engineering Division, National Institute of Standards and Technology, Gaithersburg, Maryland, United States of America; 3 Genetic Disease Research Branch, National Human Genome Research Institute, National Institutes of Health, Bethesda, Maryland, United States of America; Max Planck Institute for Evolutionary Anthropology, Germany

## Abstract

While the importance of random sequencing errors decreases at higher DNA or RNA sequencing depths, systematic sequencing errors (SSEs) dominate at high sequencing depths and can be difficult to distinguish from biological variants. These SSEs can cause base quality scores to underestimate the probability of error at certain genomic positions, resulting in false positive variant calls, particularly in mixtures such as samples with RNA editing, tumors, circulating tumor cells, bacteria, mitochondrial heteroplasmy, or pooled DNA. Most algorithms proposed for correction of SSEs require a data set used to calculate association of SSEs with various features in the reads and sequence context. This data set is typically either from a part of the data set being “recalibrated” (Genome Analysis ToolKit, or GATK) or from a separate data set with special characteristics (SysCall). Here, we combine the advantages of these approaches by adding synthetic RNA spike-in standards to human RNA, and use GATK to recalibrate base quality scores with reads mapped to the spike-in standards. Compared to conventional GATK recalibration that uses reads mapped to the genome, spike-ins improve the accuracy of Illumina base quality scores by a mean of 5 Phred-scaled quality score units, and by as much as 13 units at CpG sites. In addition, since the spike-in data used for recalibration are independent of the genome being sequenced, our method allows run-specific recalibration even for the many species without a comprehensive and accurate SNP database. We also use GATK with the spike-in standards to demonstrate that the Illumina RNA sequencing runs overestimate quality scores for AC, CC, GC, GG, and TC dinucleotides, while SOLiD has less dinucleotide SSEs but more SSEs for certain cycles. We conclude that using these DNA and RNA spike-in standards with GATK improves base quality score recalibration.

## Introduction

**Figure 1:**
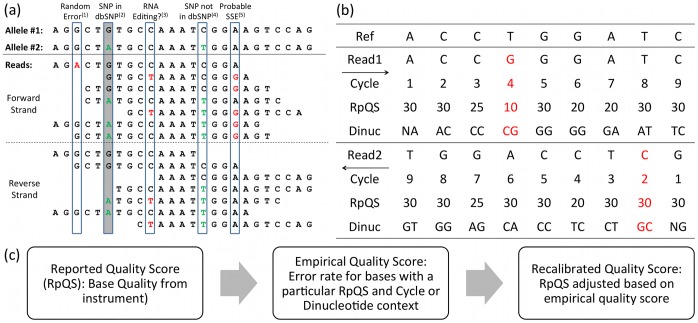
Systematic errors and base quality score recalibration. (a) Observed variants in the reads can result from a variety of biological causes and sequencing errors and biases. (1) Random sequencing errors are relatively rare at any given position in the reference, and are generally reflected accurately in the reported base quality score from the instrument. (2) Biological variants that are included in the SNP database (e.g., dbSNP for humans) are excluded from the base quality score recalibration (BQSR), and therefore do not decrease the empirical quality scores. (3) RNA editing can occur at frequencies less than 50%, so it can be difficult to distinguish from SSEs. These observed variants are treated as SSEs by the BQSR algorithm, incorrectly decreasing their base quality scores and quality scores of similar bases in other locations in the genome. (4) Biological variants that are not in dbSNP are also treated as SSEs by the BQSR algorithm, again decreasing their and similar bases’ recalibrated quality scores. (5) Since variant bases are only seen on one strand, they are likely to be SSEs. In this case, the BQSR algorithm would decrease the quality scores of the dinucleotide on the forward strand (GG). (b) Example reads and the covariates for each base used by GATK BQSR. The red columns would be counted as errors when calculating empirical quality scores. (c) Schematic of the GATK BQSR process, in which reported quality scores from the instrument are adjusted (or “recalibrated”) using empirical quality scores associated with the covariates reported quality score, machine cycle, and dinucleotide context.

As sequencing costs drop, it is becoming cost-effective to sequence even whole genomes to a sufficient depth that random errors become insignificant. However, systematic sequencing errors (SSEs) and biases remain problematic even at high sequencing depths, so recent research has started to focus on understanding these SSEs and biases [1], [2]. In this work, we focus on SSEs rather than coverage biases, where SSEs are systematic errors in sample preparation and sequencing processes that cause base call errors to accumulate preferentially at certain base positions in the genome, and coverage biases are biases in the number of reads covering certain genomic regions such as GC-bias [3]–[5]. Examples of SSEs, as well as random errors, are portrayed in Figure 1(a). Compensating for these SSEs is critical for applications in which a variant might be expected to be in only a small fraction of the reads, such as samples containing RNA-editing [6], [7], cancer tissues and circulating tumor cells [8]–[11], fetal DNA in mother’s blood [12], mixtures of bacterial strains [13], mitochondrial heteroplasmy [14], mosaic disorders [15], and pooled samples [16], [17]. Since the causes of many SSEs are not well understood and may vary due to batch effects in a run-specific manner, compensating for them requires training data sets. The two previously proposed approaches either use a separate data set with special characteristics (e.g., SysCall uses overlapping paired-end reads [1]) or use the data set itself excluding regions known to have variants (e.g., Genome Analysis Toolkit, or GATK, base quality score recalibration [2]). Here, we combine the advantages of these approaches by using DNA or RNA spike-in standards without homology to almost all biological organisms.

The first approach, SysCall, used a methyl-Seq dataset that had overlapping paired-end reads to detect SSEs depending on sequencing direction for the Illumina sequencer [1]. The region in which the reads overlap can be used to find systematic errors that preferentially occur on one DNA strand compared to the other strand. To improve variant calls, the SysCall method uses a separate dataset with overlapping reads to train a logistic regression model that accounts for SSEs correlated with several covariates: (1) the 2 preceding bases + the base in question (each base independently), (2) directionality bias of the errors, the proportion of non-reference reads, and (3) a comparison of the quality scores of the error base to the next base. Most sequencing runs do not contain overlapping paired reads, so SysCall assumes the SSEs in a training data set are the same as the SSEs in other independent sequencing runs without overlapping reads. However, even when comparing SSEs from two sequencing runs on the same sample, significant differences were found between the runs [1]. SSEs may vary even more due to constantly evolving sample preparation reagents and protocols, sequencing reagents and technologies, and bioinformatics methods. In this respect, SysCall is at a disadvantage compared to methods that perform run-specific SSE correction, such as GATK.

The second approach, GATK, is a suite of tools used for variant calling based on the map-reduce framework [2], [18]. A widely used tool implemented in GATK performs base quality score recalibration (BQSR, see Figure 1(b)). BQSR recalibrates base qualities using pre-specified and/or user-defined covariates related to the properties of the read. The covariates in BQSR are factors that are suspected to be correlated with SSEs. The most commonly used covariates implemented in GATK-BQSR are read group, base quality score, machine cycle, and dinucleotide context. BQSR calculates the empirical quality score for each combination of covariates based on the proportion of base call errors observed. Then, it compares the empirical to the reported quality scores, and recalibrates the quality scores of each base in each read depending on its corresponding covariate values. After recalibration, the base quality scores were much more predictive of the empirical quality scores [2].

One attractive feature of the GATK BQSR package is that it compensates for biases in the machine’s estimation of base calls and their quality scores by lowering the base qualities of SSEs independently in each sample, so that it can correct run- or batch-specific systematic sequencing errors. This appears to contradict established metrological practice for quantity (or numerical) values (see *Metrology for “nominal properties”* in Methods S1), where results are corrected for bias, and measurement uncertainty adjusted for propagation of error arising from the bias correction. In the absence of mature metrological guidance for nominal properties (such as sequence), adjustment of the quality score is a practical solution with the desirable property of straightforward propagation into bioinformatics pipelines such as variant calling.

In order to find run-specific SSEs, GATK BQSR typically assumes that the sample contains neither mis-mapped/mis-aligned reads nor variants (except at the bases defined by dbSNP). Since many rare variants are not in dbSNP and some SSEs might be in dbSNP [1], [19], BQSR will count many rare variants as SSEs and may miss some SSEs that are included in dbSNP (see Figure 1(a)), resulting in a bias against variants not in dbSNP. This problem is compounded for the many non-human species for which comprehensive SNP databases are not available, so many real variants will be counted as SSEs for these species. For RNA-seq, recalibrating based on dbSNP will also be biased against detecting any possible RNA editing that occurs at non-dbSNP locations. For DNA and RNA sequencing, certain motifs that are highly mutable (e.g., CpG dinucleotides) are likely to receive lower recalibrated quality scores due to incomplete dbSNP databases, resulting in variant bases receiving lower weight in the precise locations where real variants are more likely to be found. In addition, mapping errors due to closely related sequences or complex variants could cause both false positive SSEs when accurately sequenced bases are incorrectly mapped to mismatching bases, as well as false negative SSEs when inaccurately sequenced bases are incorrectly mapped to matching bases.

A few previous methods have used external spike-ins to calibrate base or variant calls. The Ibis base caller for Illumina uses the PhiX spike-in provided by Illumina to achieve more accurate base calls and quality scores by correcting machine cycle biases [20]. This method has the advantage of using the raw fluorescent intensity values in its base calling method, but the small size of the PhiX genome (≈5 kb) limits the number of covariates that can be calibrated. Another approach used short 1–2 kb external spike-in plasmids with known indels to both recalibrate quality scores and tune their parameters for detecting rare indels in large cohorts [17]. Their algorithm, called SPLINTER, used the base and the two previous bases to calibrate error rates, but they did not investigate whether the length of their short plasmids was sufficient for statistically robust calibration.

Here, we set out to test whether synthetic spike-in DNA or RNA standards with well-characterized sequence purities can be used in the GATK BQSR framework to improve detection and correction of SSEs in any sample without the need for a comprehensive and accurate SNP database.

## Results

### Synthetic Spike-in Standards

A set of 96 DNA plasmids with 273 to 2022 base pair standard sequences inserted in a ∼2800 base pair vector are a prospective NIST Standard Reference Material and a product of the External RNA Control Consortium (ERCC). The DNA plasmids were designed to be templates for RNA spike-in standards for gene expression measurements. Although the spike-in standards are generally used for quantitation, the DNA plasmids were extensively characterized by Sanger, Illumina, SOLiD2, and SOLiD3 sequencing and thus are useful for characterizing SSEs as well. These spike-in standards could be used for characterizing SSEs both in DNA and RNA sequencing, but in this paper we focus on RNA spike-ins.

### Determining High-purity Bases in Synthetic Spike-in Standard Sequences

The base quality scores reported by the instrument are frequently not accurate measures of error rates, in part due to SSEs associated with covariates such as machine cycle and dinucleotide context. Base quality score recalibration is commonly used to compensate for SSEs by adjusting base qualities, using the empirical error rates measured for bases with specific covariate values. These empirical error rates can be converted to Phred-scaled quality scores, and the difference between these empirical base quality scores and the reported base quality scores are the “recalibration coefficients” used to recalibrate the base quality scores. Ideally, only highly pure bases (*i.e.*, bases without variants) are included when calculating the empirical error rates and recalibration coefficients, so that real sequence variants are not erroneously interpreted as an SSE. Therefore, because the DNA has been extensively sequenced by the multiple methods stated above, we developed a method to determine those bases in the synthetic spike-in DNA that have >95% probability of being >99% pure. By sequencing with multiple methods and assuming that platform-specific SSEs will only cause lower purities, we determined that ≈99.95% of the bases in the spike-in standards have >95% probability of being >99% pure. Sixty percent (24/40) of the excluded bases were in the first 20 bases of the spike-in standards, where sequencing coverage was insufficient (<200) with Illumina sequencing to have >95% probability of being >99% pure, so they may in fact be of high purity, but were still excluded. The purity was estimated by examining each strand both independently and together and by using either only high quality bases or all bases, so that most of the bases with SSEs were included in the high-confidence data set. Only 8 bases in the spike-in standards had <95% purity on all platforms, and all 8 of these were excluded. These impure bases might be caused by mutations during replication of the DNA plasmids. By excluding bases that may not be highly pure, we can perform BQSR using a large set of bases (78950) in the spike-in standards for which we have >95% confidence that they are >99% pure. Note that more stringent filtering for bases with even higher purities may be necessary as sequencing technologies and their error rates improve.

### What Should the Coverage and Size of a Spike-in Standard “Genome” be for Accurate BQSR?

The 78950 highly pure bases in the spike-in standards are much fewer than in the whole genome or transcriptome, but the coverage is much higher. Therefore, it is important to understand possible inaccuracies in calculating the GATK BQSR coefficients due to the limited size of the standards. Recalibration of base quality (BQ) scores is typically based on three covariates: reported quality score (RpQS), machine cycle (position in the read), and dinucleotide context (the base and the previous base). To achieve statistically relevant recalibration scores for the large number of combinations of these covariates, recalibration must be performed using a sufficient number of bases in the reference and sufficient coverage by reads mapping to the reference. To determine whether the coverage and number of bases in the spike-in standards are sufficient, we randomly removed mapped reads and/or reference bases from the recalibration calculations, and calculated the effect on the recalibration scores.

As expected, reducing coverage or the size of the spike-in standard “genome” results in some inaccuracies in BQSR (see Figure S1 for details). For example, 1 million 100×100 bp reads mapped to the spike-in standards results in a mean error of about 0.3 and 0.2 in recalibrated quality scores for cycle and dinucleotide, respectively. For the same number of mapped reads, reducing the size of the spike-in “genome” has little effect on cycle recalibration, but significantly increases the error for dinucleotide recalibration. Therefore, accurate cycle recalibration could be performed accurately using very high coverage of a short spike-in (e.g., PhiX), but dinucleotide recalibration requires a longer spike-in such as the set of spike-in standards used in this work, which contain 78950 usable highly pure bases.

### Comparison of BQSR from Spike-in Standards vs. Whole Genome

#### Spike-in standards improve recalibration

**Figure 2:**
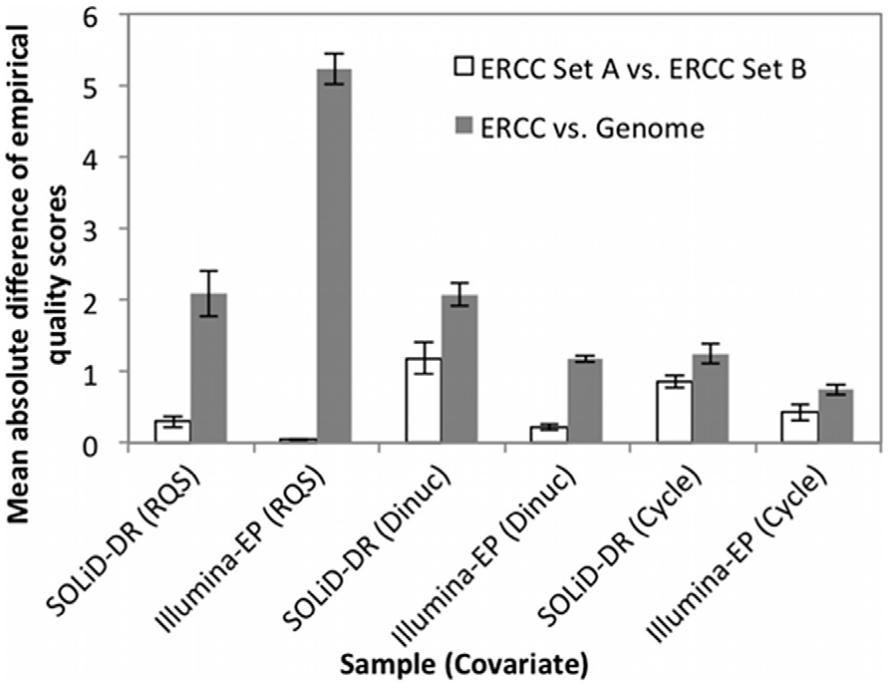
BQSR recalibration inaccuracies due to limited size and coverage of the ERCC spike-in standards, compared to the inaccuracies caused by recalibrating from the genome excluding known variant sites in dbSNP. The errors due to the limitations of the spike-in standards are the mean absolute difference between the recalibration coefficients calculated from randomly selected 50% of the spike-in standard bases (ERCC Set A) and the opposite 50% of the bases (ERCC Set B). Because the mean absolute differences are lower for the spike-in standards, they serve as a reasonable proxy for accuracy of the recalibration coefficients. Differences are calculated for the base quality score reported from the instrument (RpQS), dinucleotide context (Dinuc), and machine cycle (Cycle). The differences are the mean ± SD (n = 4) for SOLiD4 with spike-in standards spiked-in in a large dynamic concentration range with 250–700× mean coverage (SOLiD-DR), and for Illumina HiSeq with spike-in standards spiked-in at equimolar concentrations with 5500–8500× mean coverage (Illumina-EP). The use of spike-in standards for recalibration significantly improves upon the traditional genome recalibration in all cases (p<10^−4^).

To demonstrate the relative performance of BQSR using the spike-in standards compared to the typical method using the whole genome, we compared the recalibration inaccuracies caused by the limited size and coverage of the spike-in standards to the inaccuracies caused by recalibrating based on the genome. For this comparison, the 96 RNA spike-in standard sequences were spiked into human genomic RNA samples. To obtain an upper limit on the errors introduced by the limited size and coverage of the spike-in standards, half of the bases were randomly selected from the spike-in standards (spike-in set A). Then, the mean absolute difference was calculated between the recalibration values obtained from the selected bases and the recalibration values obtained from the other half of the bases in the spike-in standards (spike-in set B). In addition, the mean absolute difference was calculated between recalibrating based on the genome and recalibrating based on the spike-in standards. These calculations were performed for samples with the standards spiked-in either at equal concentrations (Illumina-EP in Figure 2) or at a large dynamic range of concentrations (SOLiD-DR in Figure 2). The p values for these differences are shown in Figure S2. Because the sequences of the spike-in standards are approximately random, the mean absolute difference between the two entirely different randomly selected sets of bases gives an estimate of the variance of the spike-in recalibration values around the true recalibration values for a random sequence. Since the mean absolute differences are significantly smaller between spike-in set A and spike-in set B than between spike-in and genome recalibration, the mean absolute differences serve as reasonable estimates of accuracy of the recalibration values for a random sequence. Biological genome sequences are not random and may contain more complex sequence motifs associated with SSEs, mapping errors, or alignment errors. However, it is likely better to perform recalibration on random sequence for simple covariates like reported quality score, dinucleotide, and cycle so that, for example, errors associated with complex sequence motifs do not get applied to all dinucleotide motifs.

The accuracy of genome recalibration was significantly reduced (p<10^−4^) for reported quality scores (RpQS), dinucleotide context (Dinuc), and machine cycle (Cycle) for both Illumina-EP and SOLiD-DR. Genome recalibration was particularly more biased for Illumina-EP and for the dinucleotide and RpQS covariates. The biases for spike-in standard recalibration were very small for Illumina-EP both because the spike-in standards were at equimolar concentrations and because they had higher coverage. However, even when spiking in standards at a wide range of concentrations, as is often done for differential gene expression measurements [21], the biases associated with genome recalibration are larger than biases associated with spike-in standard recalibration. In the next section, we examine the sources of the particularly large biases associated with recalibrating the RpQS and dinucleotide covariates based on the genome.

#### Whole genome recalibration introduces higher error rate and dinucleotide-specific biases

**Figure 3:**
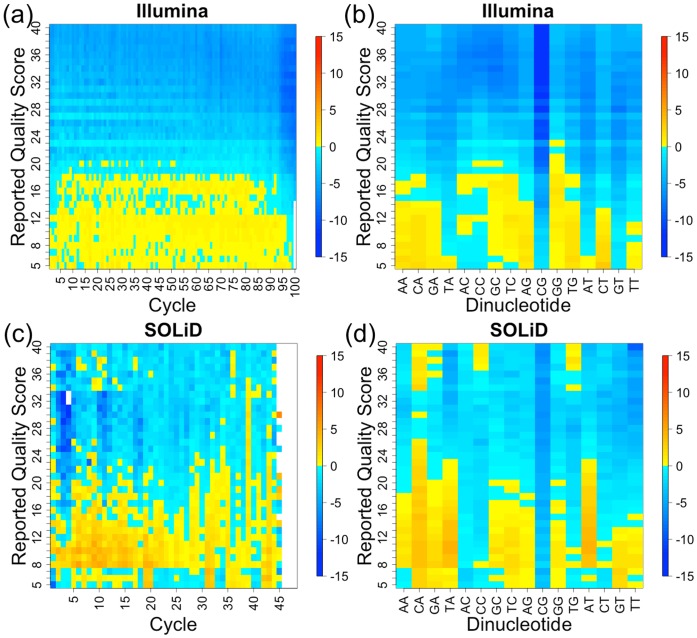
Comparison of GATK BQSR scores for recalibration based on the genome vs. recalibration based on the spike-in standards. The differences in quality score recalibration values are calculated for each combination of reported quality score and cycle (a and c) or reported quality score and dinucleotide (b and d) for Illumina (a and b) and SOLiD (c and d). White blocks correspond to very large differences, generally with very few errors. The differences are (genome – spike-in standard), so blue (<0) indicates that genome recalibration would result in recalibrated quality scores that are too low, and yellow/red (>0) results in recalibrated quality scores that are too high. The p values for the differences are shown in Figure S2.

To understand the biases caused by recalibrating based on the whole genome, we performed a statistical comparison of recalibration scores obtained from the whole genome to those obtained from the spike-in standards, with reported quality score, machine cycle, and dinucleotide context as factors (Figure 3). Two clear trends emerge: (1) the empirical error rates measured from the whole genome are generally significantly higher (empirical quality scores are an average of 5 units lower for whole genome recalibration), and (2) the CG dinucleotide in particular has a much higher error rate based on the whole genome (empirical quality scores are as much as 13 units lower for whole genome recalibration).

A likely explanation for the generally higher empirical error rates (or lower empirical quality scores) measured based on the whole genome is that dbSNP does not include all variants, especially variants found at a low population frequency. The variants not included in dbSNP, as well as non-reference bases resulting from RNA editing and mismapped reads (see Figure 1), are treated as sequencing errors, resulting in erroneously high empirical error rates. These variants also explain the large differences observed in Figure 2 between recalibrating based on the genome and recalibrating based on the spike-in standards for reported quality score. In addition, reads that map incorrectly to the whole genome can cause both false positive SSEs when accurately sequenced bases are incorrectly mapped to mismatching reference bases, as well as false negative SSEs when inaccurately sequenced bases are incorrectly mapped to matching reference bases. Incorrectly mapped reads could cause bases with high RpQS to have higher than expected empirical error rates, and bases with low RpQS to have lower than expected empirical error rates. RNA-seq reads may have even higher empirical error rates DNA-seq reads due to mismapped bases around exon-exon junctions. However, the high error rates at CG dinucleotides suggest that variants not in dbSNP also contribute to the higher error rates when calibrating based on the genome.

The particularly high error rates measured at CG dinucleotides for genome recalibration likely result from the higher mutation rate of methylated cytosines in CpG dinucleotides [22]. GATK measures the empirical error rate of the G in CpG dinucleotides, which has a high mutation rate because the opposite strand is also a CpG dinucleotide and has a C at this position. This high empirical error rate for CG dinucleotides is also evident in results published using GATK to perform BQSR for whole genome DNA sequencing (see Figure 2 in [2]). Similar to our results in which the CG error rate is high both for Illumina and SOLiD when recalibrating based on the genome, their results also show high CG error rates for DNA sequencing for all platforms, including Illumina GaIIx, Illumina HiSeq, SOLiD, and 454. Their results confirm that recalibrating based on the genome is likely also biased at CpG dinucleotides for DNA sequencing. The bias in genome recalibration at CpG dinucleotides explains the differences between recalibrating based on the genome and recalibrating based on the spike-in standards observed in Figure 2 for dinucleotide.

It is interesting that the empirical error rates for genome recalibration are significantly lower for some dinucleotides at low reported BQs. While the reason for this difference is unclear, it is possible that it could be caused by reads that are mis-mapped to nearly homologous regions that contain the base error.

### Platform-specific Biases Measured by Spike-in Standards

**Figure 4:**
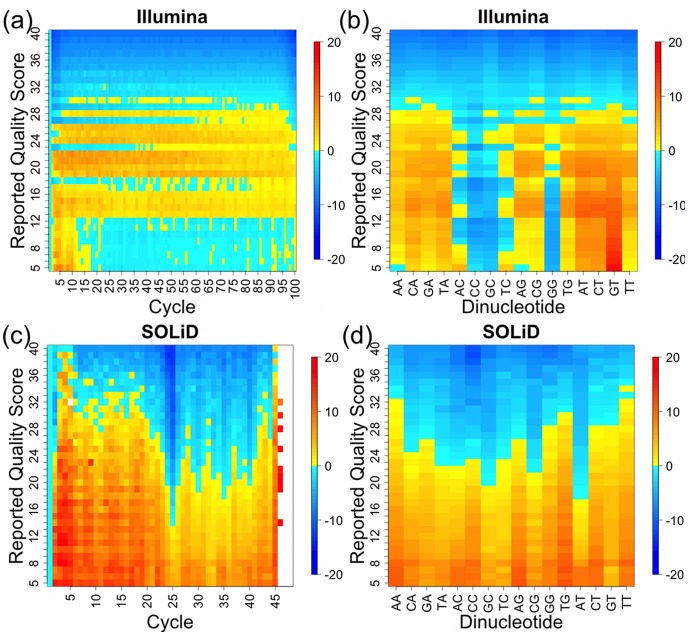
Differences between empirical and reported quality scores calculated by GATK BQSR for recalibration based on the spike-in standards. Blue (<0) indicates the reported quality scores are too high, and red/yellow (>0) indicates the reported quality scores are too low. The mean differences across all samples are calculated for each combination of reported quality score and cycle (a and c) or reported quality score and dinucleotide (b and d) for Illumina (a and b) and SOLiD (c and d), respectively.

The primary goal of the GATK BQSR algorithm is to identify the association of SSEs with covariates such as dinucleotide and cycle, and compensate for them by adjusting base quality scores. We used GATK to measure sequencing platform-specific biases by calculating the mean differences between the empirical and reported base quality scores for each combination of reported quality score and cycle, or reported quality score and dinucleotide. As shown in Figure 4, in general, the highest reported base quality scores are higher than their empirical quality scores for both instruments. However, it is difficult to measure very high empirical quality scores since even very low-level mutations in the RNA or in their reverse transcription into cDNA will lower the empirical quality scores. The mid-range reported quality scores for Illumina and the lowest reported quality scores for SOLiD tend to be lower than the empirical quality scores, which may be partly because bases not matching the reference are preferentially discarded during the mapping process. Illumina has minimal biases with respect to cycle (Figure 4a), whereas SOLiD has higher than expected empirical quality scores near the middle of the read (Figure 4c), which may result from idiosyncrasies in the way SOLiD reads are mapped by the ABI software Bioscope. In contrast, SOLiD has relatively small biases with respect to dinucleotide (Figure 4d), whereas Illumina has lower than expected empirical quality scores for AC, CC, GC, GG, and TC dinucleotides. These Illumina biases are similar the HiSeq biases observed in Figure 2d in [2], with the exception of the low CG empirical quality scores in the previous work, which likely are an artifact due to biological mutations at CG sites, as discussed above. The higher empirical quality scores for GT compared to GG dinucleotides are also consistent with the previously observed Illumina-specific SSE of GGT being mistakenly sequenced as GGG [1].

### Comparison of Nucleotide Change Errors between Recalibration Methods and Sequencing Platforms

In addition to dinucleotide and cycle biases, certain nucleotide changes have been associated with SSEs [1], DNA mutations [22], and RNA editing [6], [7]. For human DNA mutations, transitions (A<->G and C<->T) have been estimated to outnumber transversions (all other changes) by a factor of over 2 in the whole genome and over 4 in the exome. Random sequencing errors have a transition/transversion ratio (Ti/Tv) of 0.5. The C>T transition mutation is very common at CpG dinucleotides. Deaminase enzymes are known to cause A>G and C>I/T (C changes to inosine, which is read as T) transitions in RNA. T>G transversions were found to be common SSEs on the Illumina GAIIx platform [1].

**Figure 5:**
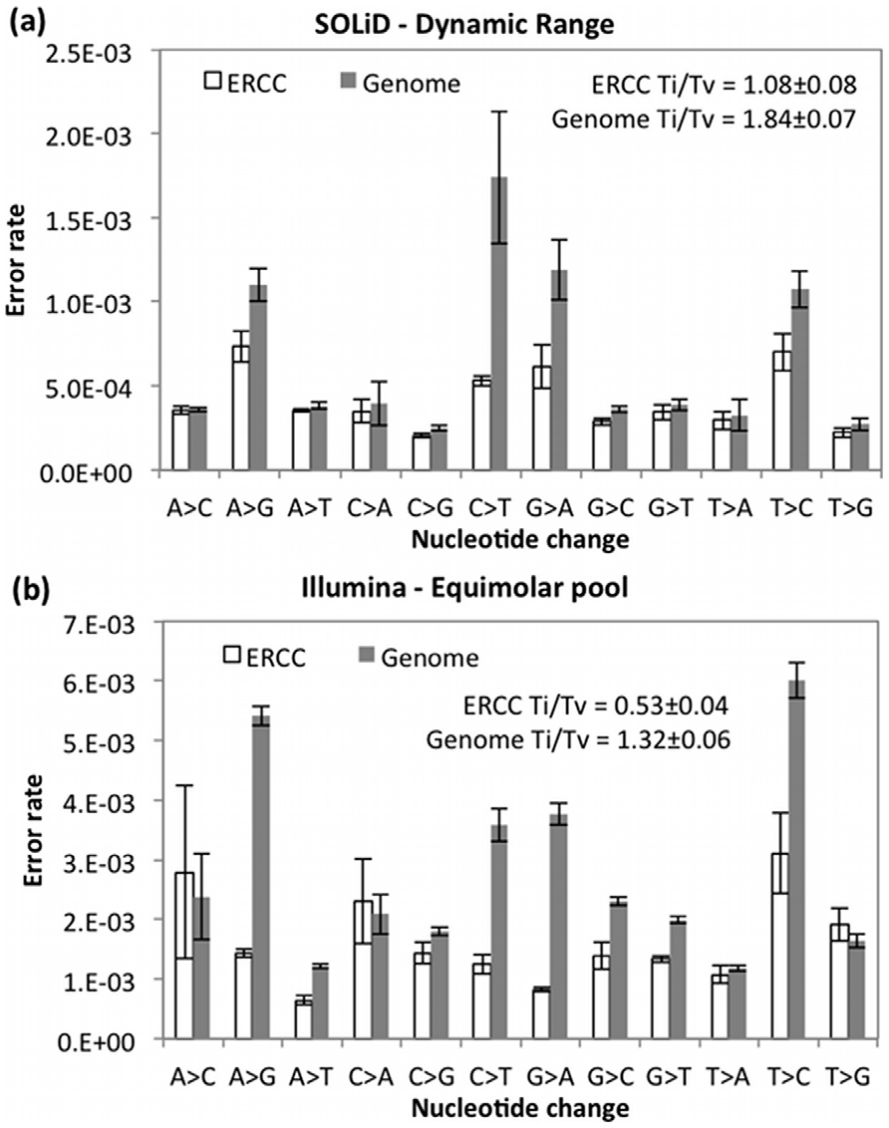
Comparison of error rates for each type of nucleotide change for spike-in standard or genome recalibration with (a) SOLiD 4 data with standards spiked-in in a large dynamic concentration range or (b) Illumina HiSeq data with standards spiked-in at equimolar concentrations. The plots are annotated with transition/transversion (Ti/Tv) ratios, where random base changes result in Ti/Tv = 0.5, and biological mutations result in Ti/Tv >>0.5. To determine the significance of biological variants in the data, only bases with reported reported base quality scores above 30 are included in this analysis. All values are the mean ± SD of 2 samples with 2 biological replicates or of 4 sequenced samples with no replicates.

We calculated the frequency of each nucleotide change for Illumina and SOLiD when recalibrating based on the genome or the standard (Figure 5). These nucleotide change error rates were the proportion of each base not matching the reference base compared to the total number of each type of base (whether it matches the reference or not). The error rates were calculated separately for the genome (excluding dbSNP sites) and spike-in standards (excluding impure bases) from the same datasets used in Figures 3, 4. To minimize random errors and thereby determine the presence of biological mutations in the data, only bases with reported BQs above 30 were included in the analysis. Because transition mutations tend to occur much more frequently than transversions, whereas sequencing errors tend to be more transversions, the Ti/Tv ratio has been used as a measure of the frequency of sequencing errors in variant calls. Therefore, we calculated the Ti/Tv ratio from the nucleotide change error rates described in Figure 5. For both SOLiD and Illumina sequencing, the Ti/Tv ratio is much higher for errors in the genome than in the spike-in standards, suggesting that a significant number of biological variants exist in the genome that are not in dbSNP. This higher Ti/Tv ratio also supports our finding in Figure 3 above, where CpG dinucleotides, which are highly mutable, have much higher error rates in the genome than in the spike-in standards.

In Figure 5, the error rates for high-quality SOLiD bases are much lower than the error rates for high-quality Illumina bases. However, it should be noted that the SOLiD mapping process can be biased towards reference calls, since colorspace errors are changed to match the reference. Therefore, the lower apparent error rate for SOLiD may result from reference bias, lower sequencing error rates, or some combination. In this way, the BQSR method can be negatively affected by reference bias, mapping errors, or any other systematic errors not included as covariates in the recalibration process.

## Discussion

In this work, we have demonstrated the utility of synthetic spike-in standards to interrogate run-specific SSEs in DNA or RNA sequencing. These standards can be used within the GATK framework to recalibrate base quality scores when sequencing species that do not have a comprehensive SNP database. Even for human DNA, which has a very large SNP database, rare variants cause significant biases in the typical GATK recalibration process, which uses reads mapped to genomic bases not in dbSNP. These biases are particularly large for CpG dinucleotides, which have a high mutation rate, so GATK recalibration without spike-in standards significantly degrades variant base qualities at the base positions where variants are much more likely to occur.

It should be noted that some of the differences between recalibration based on the spike-in standards vs. based on the genome could result from SSEs or mapping errors in complex CpG-related sequence motifs in the genome that are not present in the spike-in standards. However, for this to be true, these sequencing motifs would have to cause the same SSEs in multiple sequencing platforms, since CpG dinucleotides have very low empirical quality scores for both Illumina and SOLiD in this work, and for Illumina GAIIx, Illumina HiSeq, SOLiD, and 454 in previous work [2]. In addition, if complex sequencing motifs cause SSEs, then an algorithm should ideally penalize only the complex sequence motifs causing the SSEs, rather than penalizing all CpG dinucleotides. Future work should be dedicated towards identifying more complex sequence motifs and other covariates associated with SSEs, which could be incorporated into GATK.

By using synthetic spike-in standards, the mean errors in recalibrated quality scores (for RpQS, cycle, and dinucleotide) can be significantly decreased to <0.5 (in quality score units) for 5 million 100-bp reads (or about 0.5% of a 30× human whole genome sequencing run). For RNA-sequencing, the synthetic spike-in standards significantly reduce biases even when spiked-in at a large dynamic range of concentrations for differential expression analysis and using less than 10^6^ 50-bp reads, and biases can be reduced even further when using the spiked-ins at equimolar concentrations and at higher concentrations. In summary, we have demonstrated that adding synthetic spike-in standards can improve SSE recalibration within the current GATK framework for human sequencing, and they allow SSE recalibration for species without comprehensive SNP databases, even when only a small fraction of the total reads is dedicated to the synthetic spike-ins.

## Materials and Methods

### ERCC Spike-in Standards

The candidate NIST Standard Reference Material (SRM) 2374 DNA plasmids were used for DNA sequencing. These plasmids consist of ∼2800 bases of vector sequence with 273–2022 base standard sequence inserted in the vector. In addition, libraries of RNA were prepared from the 96 DNA plasmids in this SRM by *in vitro* transcription and pooled either at equimolar concentrations (for Illumina sequencing) or at a large ≈10^6^ dynamic range of concentrations (for SOLiD sequencing), as described in Methods S1 and Supporting Information S1 - NIST Prepared RNA pools from SRM 2374.xlsx.

### Preparation of DNA Spike-in Standards for Sequencing

Sequencing of the DNA spike-in standards was performed on Illumina GA, SOLiD v2, and SOLiD v3+ using standard methods described in detail in Methods S1. On Illumina GA, ≈9.1 million single-end 39-bp reads from the pooled 96 ERCC spike-in standards, 10 of which contained the vector. On SOLiD 2, ≈70 million single-end 35-bp reads from the pooled 96 ERCC spike-in standards, all of which contained the vector. On SOLiD 3+, ≈420 million 50×50-bp mate-pair reads (mean insert size of ≈530 bp) from the pooled 96 ERCC spike-in standards, all of which contained the vector.

### Preparation of RNA BLM Libraries with Spike-in Standards for Sequencing

Mixtures of RNA from brain, liver, and muscle tissues were prepared, similar to those described previously [23]. A large dynamic concentration range pool of ERCC spike-in standard RNA was added at 3% of the total RNA concentration, resulting in approximately 10% of the total reads coming from the spike-ins. Bar-coded RNA sequencing on SOLiD 4 was performed as described in Methods S1, resulting in 5 to 15 million 50-bp single-end reads from each sample (GEO Accession Number GSE36217).

### Cell Line Establishment and Cell Culture for Illumina RNA Sequencing

Lymphoblastoid cell lines (LCLs) were established from four human male subjects of Caucasian origin by Epstein-Barr Virus transformation of B-lymphocytes using standard procedures [24]. For each of these LCLs, culture was seeded with 10^6^ cells in 5 ml of RMPI1640 (Invitrogen, CA) with 20% Fetal Bovine Serum (Invitrogen, CA), incubated at 37°C with 5% CO_2_ and grown at a density of 2–3×10^5^ cells/ml. Cells were harvested when the culture reached 10^7^ cells in total, washed with 50 ml of 1X PBS (Invitrogen, CA) and frozen at −80°C in aliquots of 4×10^6^ cells for downstream processing. These cell lines were established with written informed consent from the human subjects with approval from the National Institutes of Health Institutional Review Board under the ClinSeq project.

### RNA-Seq Library Preparation and Illumina Sequencing

Total RNA was extracted using the RNeasy Mini kit (Qiagen, MD). RNA integrity was assessed using the Agilent 2100 Bioanalyzer RNA 6000 Nano Assay (Agilent, CA). All samples had RIN (RNA Integrity Number) higher than 9.5 as measured by this assay. For each cell line, aliquots of 5 micrograms of total RNA were spiked with 3% by weight of an equimolar mix of the ERCC spike-in standards and RNA-Seq libraries were constructed using a modified version of the technique described in Marioni et al (2008). The following notable changes were made to their protocol: RNA fragmentation was performed using the Covaris S2 AFA platform (Covaris, MA) and size selection of adapter-ligated cDNA was done using the Pippin Prep instrument (Sage Science, MA). RNA-Seq libraries were sequenced on an Illumina GAII_x_ DNA sequencer (Illumina, CA). Raw image data generated by the sequencer was processed using Illumina RTA software (version 1.8.70.0). One paired-end 101 bp lane of data was generated for each library, resulting in ≈100 million reads from each of the four samples (GEO Accession Number GSE36217).

### Determining Bases in Spike-in Standards with >95% Probability of >99% Purity

To determine which bases in the spike-in standards to include in the BQSR, we used a Bayesian statistical model to calculate which bases had a >95% probability of having a purity ≥99%. Our statistical model is described in detail in Methods S1, with associated Perl and Matlab scripts in Code S1 - PileupParseQualFilt.pl, Code S2 - calcpurpropthresh.m, and Code S3 - PileupSummaryPurityProbThreshBases.m. A vcf with the excluded bases is Supporting Information S2 - ercclowpur99skip50basepolyA.vcf.

### Determining Optimal Coverage of Spike-in Standards and Effect of Recalibrating on a Shorter Sequence

To determine the effect of coverage on the recalibration values, the mapped F3 reads in the bam file from the mate-pair library (MP) were downsampled using the Picard (v. 1.5) tool DownsampleSam.java, which randomly removes reads from the bam file. The reads were downsampled by factors of 3, 9, and 27, with the factor of 27 resulting in coverage of the spike-in standards similar to the Illumina and SOLiD2 libraries.

Similarly, one might want to know whether a smaller set of spike-in standards (*e.g.,* PhiX) could be used to perform recalibration. Therefore, to determine the effect of recalibrating from shorter sequences (i.e., fewer bases in the “genome”), random bases were excluded from the GATK analysis by adding them to the vcf file (normally used by GATK to exclude bases with known biological variation). Between 10% and 90% of the high-purity ERCC spike-in standard reference bases were randomly selected using the *sample* function in R, and then added to the vcf file for GATK analysis. The CountCovariates method in GATK (v. 1.2–62) was used to calculate the recalibration values for each combination of covariates, using read group, reported quality score, dinucleotide, and machine cycle as covariates, and with default parameters except with the options –solid_recal_mode REMOVE_REF_BIAS and –solid_nocall_strategy PURGE_READ.

To be consistent with how GATK performs recalibration, we aggregated the recalibration values by reported quality score and cycle or by reported quality score and dinucleotide, and then calculated the weighted mean absolute differences between the downsampled recalibration values and the original recalibration values (without downsampling). The values were weighted by the number of observations in each group to obtain the expected mean error in recalibration due to downsampling.

### Errors Associated with Genome Recalibration and Limited Length/coverage of Spike-in Standards

For all RNA-sequencing data, reads were mapped to the hg18 human reference with the ERCC spike-in standard sequences appended. Reads from SOLiD 3+ were mapped using the Whole Transcriptome pipeline in Bioscope (v. 1.3) with default parameters. Reads from Illumina HiSeq were mapped using BWA (v. 0.5.9) with default parameters. The resulting bam files were split into two bam files with reads mapping to hg18 or with reads mapping to the spike-in standards using the Picard tool ReorderSam. GATK was used with the same parameters as described above, except with the dbSNP v.132 vcf file (downloaded from the GATK website) for reads mapped to hg18, and with the modified vcf files containing randomly selected 50% of the bases in the spike-in standards, as described in Methods S1. Differences between BQSR recalibration based on the genome vs. based on the spike-in standards were calculated for each combination of reported quality score and machine cycle or reported quality score and dinucleotide, similar to the GATK BQSR process. Similarly, the differences between the empirical quality scores calculated based on the spike-in standards and the reported quality score were calculated. More detailed methods and their associated scripts are provided in Methods S1. The statistical calculations were performed using the R scripts Code S4 - makeData.R, Code S5 - analysis.R, and Code S6 - QempvQrep.R for Illumina; Code S7 - makeDataWT.R, Code S8 - analysisWT.R, and Code S9 - QempvQrepWT.R for SOLiD; and Code S10 - biasAgg.R.

### Comparison of Nucleotide Change Errors between Recalibration Methods and Sequencing Platforms

To calculate error rates for each type of nucleotide change, we used custom Perl scripts (included in the supporting information) to parse the samtools (v. 0.1.13) pileup output, calculating the error rates for each type of nucleotide change, excluding bases in dbSNP (v. 132) from the genome, and only including high purity bases in the spike-in standards. To minimize the influence of random errors, only bases with reported BQ scores of at least 30 were included in this analysis. For each nucleotide change, the mean errors using ERCCs or the genome for recalibration were statistically compared using paired t-tests with a pooled variance, accounting for multiple comparisons with a Bonferroni adjustment.

### Sequencing Data Accession Numbers

Sequencing data has been submitted to the Short Read Archive at NCBI via GEO, with accession numbers *submitted during review.*

## Supporting Information

Figure S1 **Effects of decreasing coverage and/or number of bases in the recalibration reference on (a) cycle and (b) dinucleotide recalibration values.** To decrease coverage, reads were randomly downsampled to 33%, 11%, or 3.7% of the total mapped reads, and empirical quality scores were calculated. To decrease bases in the reference, 0% to 90% of the bases (in increment of 10%) in the spike-in standard were randomly removed from the calculations of the empirical quality scores. The mean absolute difference of the empirical quality scores (i.e., the difference between using all bases/reads and using a subset of bases and/or reads) was calculated from high-coverage (≈50000× mean) SOLiD3+ mate-pair sequencing of the 78950 highly pure bases in the DNA spike-in standards. Decreasing the size of the reference has a similar effect as decreasing the coverage on the cycle recalibration scores, but is more deleterious for the dinucleotide recalibration scores.
(TIF)

Figure S2 **Statistical comparison of GATK BQSR scores for recalibration based on the genome vs.** recalibration based on the standards in Fig. 3. The p values are calculated using the multivariate logistic regression model described in Supporting Methods S1 and compensated for multiple comparisons.
(TIF)

Methods S1 **Detailed methods for library preparation and statistics.**
(DOCX)

Supporting Information S1 **concentrations of each of the ERCC spike-ins.**
(XLSX)

Supporting Information S2 **vcf with ERCC spike-in bases excluded from GATK analysis.**
(VCF)

Code S1 **Pileup parsing script.**
(PL) Click here for additional data file.

Code S2 **Matlab script to calculate purity probability thresholds for spike-in bases.**
(M) Click here for additional data file.

Code S3 **Matlab script to calculate purity probability thresholds for spike-in bases.**
(M) Click here for additional data file.

Code S4 **R script to import Illumina BQSR data.**
(R) Click here for additional data file.

Code S5 **R script to analyze Illumina BQSR data.**
(R) Click here for additional data file.

Code S6 **R script to plot Illumina BQSR data.**
(R) Click here for additional data file.

Code S7 **R script to import SOLiD BQSR data.**
(R) Click here for additional data file.

Code S8 **R script to analyze SOLiD BQSR data.**
(R) Click here for additional data file.

Code S9 **R script to plot SOLiD BQSR data.**
(R) Click here for additional data file.

Code S10 **R script to aggregate BQSR data.**
(R) Click here for additional data file.
