# Supplementary figures and images for: Synthetic Spike-in Standards Improve Run-Specific Systematic Error Analysis for DNA and RNA Sequencing
Source: PLoS One. 2012 Jul 31;7(7):e41356. doi: 10.1371/journal.pone.0041356 (PMC3409179; doi:10.1371/journal.pone.0041356)

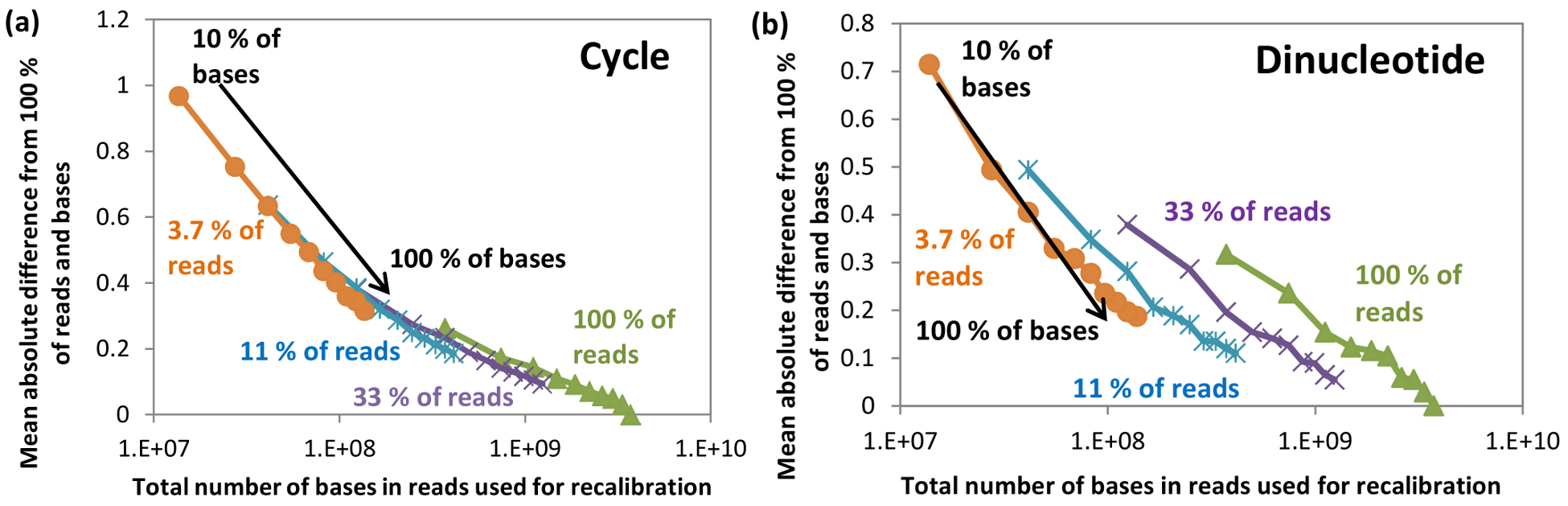

Supplement: Figure S1 — Effects of decreasing coverage and/or number of bases in the recalibration reference on (a) cycle and (b) dinucleotide recalibration values. To decrease coverage, reads were randomly downsampled to 33%, 11%, or 3.7% of the total mapped reads, and empirical quality scores were calculated. To decrease bases in the reference, 0% to 90% of the bases (in increment of 10%) in the spike-in standard were randomly removed from the calculations of the empirical quality scores. The mean absolute difference of the empirical quality scores (i.e., the difference between using all bases/reads and using a subset of bases and/or reads) was calculated from high-coverage (≈50000× mean) SOLiD3+ mate-pair sequencing of the 78950 highly pure bases in the DNA spike-in standards. Decreasing the size of the reference has a similar effect as decreasing the coverage on the cycle recalibration scores, but is more deleterious for the dinucleotide recalibration scores. (TIF) [file pone.0041356.s001.tif]

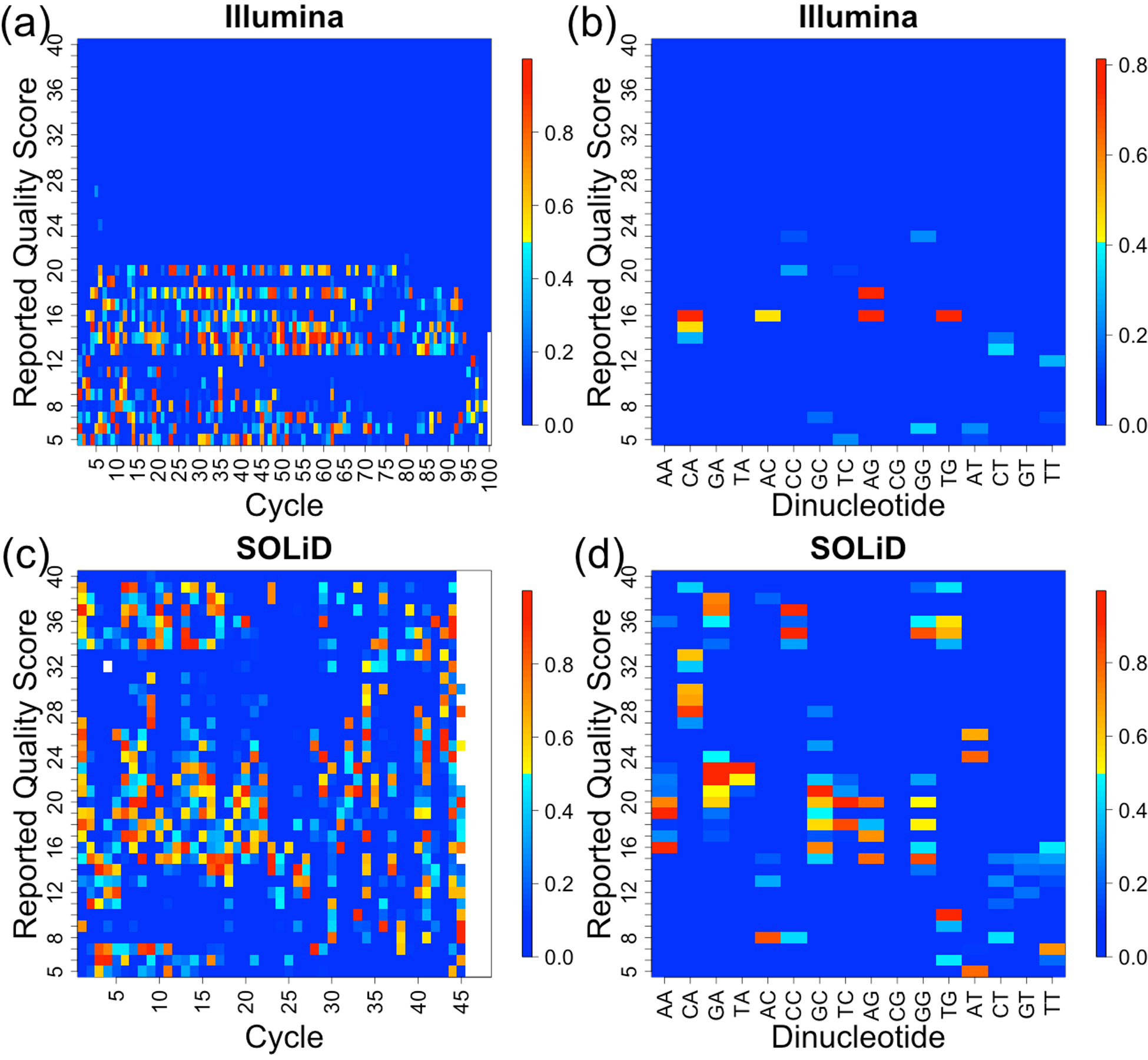

Supplement: Figure S2 — Statistical comparison of GATK BQSR scores for recalibration based on the genome vs. recalibration based on the standards in Fig. 3. The p values are calculated using the multivariate logistic regression model described in Supporting Methods S1 and compensated for multiple comparisons. (TIF) [file pone.0041356.s002.tif]
