# Supplementary material for: Synthetic Spike-in Standards Improve Run-Specific Systematic Error Analysis for DNA and RNA Sequencing
Source: PLoS One. 2012 Jul 31;7(7):e41356. doi: 10.1371/journal.pone.0041356 (PMC3409179; doi:10.1371/journal.pone.0041356)
Supplement: Methods S1 — Detailed methods for library preparation and statistics. (DOCX) [file pone.0041356.s003.docx]

**Supporting Methods S1 for “Synthetic spike-in standards improve run-specific systematic error analysis for DNA and RNA sequencing”**

# *Metrology for “nominal properties”*

Metrology does not yet have a coherent and well-posed set of tools (such as metrological traceability and measurement uncertainty for quantity values) for working with so-called “nominal properties” (a property that can take on a set of values that cannot be ordered or operated upon algebraically) like sequence. In practice, confidence in sequence measurements has been expressed as a log-scale probability of error (“Q” or “quality score”), which is roughly analogous to measurement uncertainty for quantity values. Therefore, correction for systematic sequencing errors (SSEs) has been done by adjusting the quality score.

# *Pooling strategy for SOLiD BLM1+ and BLM2+ RNA mixtures with spike-in standards*

Table S1 shows the basic pool 1.5 fold pooling strategy used for BLM1+ and BLM2+.   The first column is the 3 subpools of ERCC transcripts.  Each subpool is composed of 32 different ERCCs.  Within each subpool the log 2 relative abundance of the controls spanned approximately 2^20^.  The concentrations of each of the ERCC transcipts are shown in Supporting Information S1 - NIST Prepared RNA pools from SRM 2374.xlsx.

The BLM pools were mixed using the ratios of Muscle:Liver:Brain shown below and then the ERCC subpools A, B and C were mixed in the same way.  Brain and Subpool C remains constant in each pool.  The BLM pools were then spiked with pool 78A or 78B prior to ribodepletion.  We have empirically determined that adding the spike-in standards at 3 % of total RNA results in approximately 10 % of the sequencing reads mapping to the spike-in standards. .

BLM1+ = BLM mix Pool-A spiked with ERCC subpool Pool-A

BLM2+ = BLM mix Pool-B spiked with ERCC subpool Pool-B

Table S1: Pooling fractions for brain, liver, and muscle libraries with ERCC spike-in standards

|  | Pool-A Fractions | Pool- B Fractions |
| --- | --- | --- |
| A/ Muscle | 0.45 | 0.30 |
| B/ Liver | 0.30 | 0.45 |
| C/ Brain | 0.25 | 0.25 |

# *SOLiD Library and bead preparation for RNA sequencing*

ERCC spike pool 78A and 78B were combined with BLM Pool-A and BLM Pool-B respectively to generate samples BLM1+ and BLM2+. Five micrograms of each total RNA plus spike-in sample was ribodepleted using the RiboMinus™ Human/Mouse Transcriptome Isolation Kit, Catalog Number K1550-01 (Invitrogen, Carlsbad, CA). Approximately 400 ng of ribodepleted RNA in solution was converted to cDNA libraries with SOLiD™ adaptors and barcodes, using the SOLiD™ Total RNA-Seq Kit, PN 4445374, and protocol PN4452437 Rev. A (Life Technologies, Carlsbad, CA). Ribodepleted RNA was enzymatically fragmented with RNase III using the stated protocol methods and assessed on a 2100 Bioanalyzer (Agilent Technologies, Santa Clara, CA) with the RNA 6000 Pico Kit (Agilent Technologies, Santa Clara, CA). Fragment distributions were found to have a median size of approximately 125 base pairs.  SOLiD™ adaptors were then ligated onto the RNA and the RNA was reverse transcribed. cDNA products were then amplified and uniquely barcoded using the [SOLiD™ RNA Barcoding Kit, Modules 17-32](https://products.appliedbiosystems.com:443/ab/en/US/adirect/ab?cmd=catProductDetail&productID=4453189&catID=606680&backButton=true) (Life Technologies, Carlsbad, CA). Following barcoding and amplification, completed cDNA libraries were assessed for size distribution and yield on the 2100 Bioanalyzer with the DNA 1000 Kit (Agilent Technologies, Santa Clara, CA). A smear analysis was performed and confirmed <21% of resulting cDNA was smaller than 200 nucleotides and was appropriate for templated bead preparation. Individual cDNA libraries were quantified using qPCR on a 7900 HT Real-Time PCR System (Life Technologies, Carlsbad, CA) with the SOLiD™ Library TaqMan® Quantitation Kit, PN 4449639 (Life Technologies, Carlsbad, CA) and pooled with other barcoded samples to create an equimolar pool. Pooled libraries were quantified using qPCR to confirm final molar concentration for use in templated bead preparation. Templated beads were prepared by emulsion PCR (ePCR) using the Applied Biosystems EZ Bead System (Life Technologies, Carlsbad, CA). Two library titration points, 0.4 and 0.8 pM, were initially used for work flow analysis to determine input for full ePCR. Upon completion of work flow analysis run on an Applied Biosystems SOLiD™ v4 platform (Life Technologies, Carlsbad, CA) it was determined that a 0.6 pM titration for library input would be used in templated bead preparation to achieve maximum yield of high quality (≥ 77% On-Axis, ≤7% Noise to Signal) P2 enriched beads. Two rounds of templated beads were prepared for measurement on the SOLiD™ v4 instrument.  Samples were run in two separate quads on each of two flow cells of an Applied Biosystems SOLiD™ v4 instrument using ToP chemistry to generate paired-end reads of 50 base pairs forward, 35 base pairs reverse and a 10 base pair barcode.  

*Determining bases in spike-in standards with >95 % probability of >99 % purity*

To determine which bases in the spike-in standards to include in the BQSR, we used a Bayesian statistical model to calculate which bases had a > 95 % probability of having a purity ≥ 99 %. This Bayesian model is similar to the model in bam2mpg (http://research.nhgri.nih.gov/software/bam2mpg/) that calculates the genotype probability (which assumes the location is homozygous or heterozygous), but our model calculates the probability distribution of purities instead of a genotype probability:

$P\left( H_{x} | D \right)=\frac{P\left( D | H_{x} \right)P{(H}_{x})}{\int_{0}^{1} P\left( D | H_{x,i} \right)P{(H}_{x,i})dH_{x}}$ (1)

Where H_x_ is the hypothesis that the proportion (or purity) of base x = p_Hx_ and D is the observed data (i.e., k_x_ of the total reads r support base x, where r = coverage). We do not use prior information about the purity probability distribution (i.e., “flat prior”), so P(H_x_) = 1.

P(D|H_x_) is expected to follow the binomial distribution:

$P\left( D | H_{x} \right)=\binom{r}{k_{x}}{p_{Hx,corr}}^{k_{x}}{(1-p_{Hx,corr})}^{r-k_{x}}$ (2a)

$p_{Hx,corr}=p_{Hx}\left( 1-\epsilon\right)+(1-p_{Hx})\epsilon$ (2b)

Where p_Hx,corr_ is the expected proportion of reads that support base x corrected for the sequencing error rate ε. ε = 0.001 was used here as a low conservative estimate since the empirical quality scores calculated by GATK were generally around 30 or less. For our application, the probability distribution is not very sensitive to ε since the useful measured purities are generally greater than the minimum purity of interest. To enable computation for very large r > 1000, Eq. 2a is approximated as a normal distribution using the de Moivre–Laplace theorem.

To calculate the probabilities of each purity, the integral in the denominator is discretized, each term is calculated (for p_Hx_ from 0 to 1), and then each is normalized by the sum to get P(H_x_|D). To find the probability that the purity ≥ 99 %, P(H_x_|D) is integrated over purities ≥ 99 %.

To minimize contributions from instrument-specific SSEs, three independent HTS measurements were performed on the ERCC spike-in SRM, as described above and in the supporting information. The three high-coverage HTS runs were used to determine bases with a >95% probability of having >99% purity. In addition, to minimize artifacts from strand-biased SSEs, reads from each strand are considered both independently and combined. Finally, to minimize contributions from bases with low BQs, this calculation was performed both for all bases and only for bases with BQ > 30 for Illumina and BQ > 50 for SOLiD. We assumed that instrument-specific SSEs were unlikely to cause the purity to be overestimated, so if either strand on any of the three runs (with or without quality filtering) predicts a probability > 95 % that the base purity ≥ 99 %, then that base was considered highly pure.

Both Illumina and SOLiD reads were mapped to a reference derived from capillary sequencing for each of the ERCC spike-in standards (without the poly-A tails) and to the vector sequence. Mapping was performed using BFAST 0.65a with the default parameters and the recommended 8 seeds for Illumina and 10 seeds for SOLiD for most sensitive mapping. We used the option -a 3 for multiply mapped reads, so that only the mapping location with the highest mapping quality was kept, and reads that had multiple optimal mapping locations were rejected to avoid ambiguities. Because the ERCC sequences were designed to minimize similarity among sequences, very few mapping ambiguities existed, and less than 1 in 100000 reads had multiple optimal mapping locations even for short 35-bp SOLiD 2 reads. The mapped reads were then converted into a pileup by the mpileup tool in samtools, and the mpileup file was parsed using custom perl (Supporting Code S1 - PileupParseQualFilt.pl) and Matlab scripts (Supporting Code S2 - calcupurpropthresh.m and Supporting Code S3 - PileupSummaryPurityProbThreshBases.m) to determine the purities of each base.

In addition, to avoid mapping ambiguities around the poly-A tail, the poly-A tail and the previous 50 bases were excluded from all analyses in this work. A vcf with the excluded bases is Supporting Information S2 - ercclowpur99skip50basepolyA.vcf.

*Errors associated with genome recalibration and limited length/coverage of spike-in standards*

It is important to recognize that the mapping algorithm and its parameters can have a significant effect on the empirical quality scores, because higher stringency mapping results in fewer errors being included in the mapped reads. Therefore, the same mapping algorithm and parameters should be used to map the reads both to the spike-in standards and to the genome to be recalibrated.

To calculate the errors associated with the limited length and coverage of the spike-in standards, we randomly selected half of the bases from the spike-in standards (ERCC Set A) and excluded them from the GATK analysis using the vcf file as described in the Methods. Then, we excluded the opposite half of bases from the GATK analysis (ERCC Set B), and compared the resulting recalibration values. Similar to the recalibration process described in the GATK paper (DePristo et al. 2011), we used R (v. 2.12.1) first to aggregate observations and mismatches in the GATK recalibration table by reported quality score (RpQS), resulting in a difference between empirical and reported quality scores for each reported quality score (ΔQ(r) in (DePristo et al. 2011)). Then, we aggregated by reported quality score and dinucleotide, and subtracted ΔQ(r), resulting in a dinucleotide-specific difference between empirical and reported quality scores for each dinucleotide and RpQS (ΔQ(r,d) in (DePristo et al. 2011)). Similarly, we aggregated by reported quality score and machine cycle, and subtracted ΔQ(r), resulting in a cycle-specific difference between empirical and reported quality scores for each machine cycle and RpQS (ΔQ(r,c) in (DePristo et al. 2011)). We randomly created 20 pairs of A and B data sets from each sequencing run and computed the mean and standard deviation of the differences between sets A and B. These variances between the 20 pairs of data sets from a single sequencing run were generally much smaller than the variance between different runs, so the variance between runs is reported. For both the Illumina and SOLiD sequencing datasets, the mean of the difference between ERCC sets A and B was compared to the mean of the difference between the genome and ERCC recalibration sets, independently for RpQS, cycle, and dinucleotide. The statistical comparison was performed using paired t-tests with a pooled variance, accounting for multiple comparisons using a Bonferroni adjustment.

*Statistical comparison of recalibration based on standards vs. genome*

For the same SOLiD and Illumina RNA-sequencing runs, we performed a statistical comparison of BQSR recalibration values based on standards vs. genome for each combination of reported quality scores and cycles or dinucleotides. In our analysis of the relationship between the genome and spike-in standard recalibration methods we begin by “aggregating” the total and mismatched reads over reported quality score (RpQS) by dinucleotide and machine cycle number (i.e., position in the read), respectively. By aggregating we simply mean that for each unique RpQS, we sum over the total and mismatched reads for a particular dinucleotide (or cycle). So, for example, if we were looking at the *i^th^* RpQS with dinucleotide *j*, cycle *k*, RNA prep *l* and calibration method *r* then corresponding­ total and mismatched reads would be *t_i_*_­_*_­jklr ­_*and *m­­_ijklr_*. Thus, aggregating over cycle we would have­­

$c_{ijlr}^{t}=\sum_{k=1}^{n_{cycle}} t_{ijklr} \mathrm{and}$ $c_{ijlr}^{m}=\sum_{k=1}^{n_{cycle}} m_{ijklr},$

and similarly summing over dinucleotide would give us $d_{iklr}^{t}$ and $d_{iklr}^{m}$. Here *n_cycle_* is the number of cycles (*n_dinucleotide_* is the number dinucleotides) and $c_{ijlr}^{t}$ and $c_{ijlr}^{m}$ correspond to the total and mismatched reads aggregated over cycle, respectively (similar notation holds for $d_{iklr}^{t}$ and $d_{iklr}^{m}$).

Based on the nature of the data a sensible model choice is the multivariate logistic regression (McCullagh and Nelder 1989). Looking separately at each RpQS and corresponding cycle (or dinucleotide) we use a logistic regression to model the probability of a mismatch as a function of RNA preparation and calibration method. Our justification for considering each RpQS and cycle (or dinucleotide) subgroup separately is the following; while there is a clear relationship between RpQS and cycle (at the beginning and end cycles we see an increasing and decreasing trend in RpQS, respectively), by blocking RpQS and cycle we are able to control for this dependence and focus on the differences between calibration methods. Similarly, for RpQS and dinucleotide, while there are apparent dependencies, by considering each separately we are able to account for these. This also eases the interpretability of the resulting contrasts between calibration methods.

Recall that the primary question of interest revolves around the null hypothesis that no differences exist between the genome and spike-in standard recalibration methods. Because each separate analysis described above is part of this hypothesis our choice to reject (or not reject) the null needs to reflect the number of contrasts we are making, i.e. we need to ensure that our test accounts for the multiple comparisons that are being made. In order to account for multiple comparisons we use a Bonferroni adjustment. Note that separate analyses are performed on the data sets aggregated by cycle and dinucleotide respectively. The statistical calculations were performed using the R scripts Supporting Code S4 - makeData.R, Supporting Code S5 - analysis.R, and Supporting Code S6 - QempvQrep.R for Illumina; Supporting Code S7 - makeDataWT.R, Supporting Code S8 - analysisWT.R, and Supporting Code S9 - QempvQrepWT.R for SOLiD; and Supporting Code S10 - biasAgg.R.

*Effects of coverage and of the limited length of the control sequences on BQSR*

Supporting Figure S1 depicts the errors in the BQSR scores for cycle and dinucleotide that are introduced by removing reads and/or reference bases from a very high coverage DNA sequencing run. For recalibration by cycle, the errors caused by reducing coverage are similar to the errors caused by removing reference bases (i.e., decreasing coverage by a factor of 3 or reducing the number of bases in the reference by a factor of 3 both result in a mean absolute BQ recalibration error of about 0.1 in recalibrated quality score). Therefore, sufficient bases exist in the reference to recalibrate by cycle, which is expected because machine cycle recalibration scores are unlikely to be affected by reference context. However, for recalibration by dinucleotide, the errors caused by reducing coverage are significantly smaller than the errors caused by removing reference bases (i.e., decreasing coverage by a factor of 3 results in an error of 0.05, but reducing the number of bases in the reference by a factor of 3 results in an error of about 0.14). Therefore, dinucleotide recalibration requires more bases in the reference sequence because SSEs tend to accumulate at certain bases in the reference, and it is important to determine the significance of biases due to the limited size of the spike-
